# Relationship between Serum 25OH-Vitamin D2 Level and Vitamin D Status of Children Aged 3–5 Years in China

**DOI:** 10.3390/nu13114135

**Published:** 2021-11-19

**Authors:** Xuehong Pang, Zhenyu Yang, Jie Wang, Yifan Duan, Liyun Zhao, Dongmei Yu, Jianqiang Lai

**Affiliations:** Key Laboratory of Trace Element Nutrition of National Health Commission, National Institute for Nutrition and Health, Chinese Center for Disease Control and Prevention, Beijing 100050, China; pangxh@ninh.chinacdc.cn (X.P.); wangjie@ninh.chinacdc.cn (J.W.); duanyf@ninh.chinacdc.cn (Y.D.); zhaoly@ninh.chinacdc.cn (L.Z.); yudm@ninh.chinacdc.cn (D.Y.); laijq@ninh.chinacdc.cn (J.L.)

**Keywords:** China, cross-sectional study, preschool children, 25-hydroxvitamin D2, 25-hydroxvitamin D

## Abstract

Background: Vitamin D deficiency is prevalent globally and there is lack of evidence as to how 25(OH)D2 contributes to vitamin D status. The aim of this study was to describe vitamin D status and to assess the role of vitamin D2, a dietary vitamin D source, against the vitamin D status of children aged 3–5 years in China. Methods: Data were extracted from the Chinese National Nutrition and Health Surveillance (CNNHS) in 2013. The concentration of serum 25(OH)D2 and 25(OH)D3 was measured by using LC-MS/MS. Results: A total of 1435 subjects were enrolled and serum 25(OH)D were analyzed. The prevalence of total serum 25(OH)D < 30 nmol/L was 8.9%. Serum 25(OH)D2 was detected in 10.9% of the studied children. After adjusting for confounding factors, total 25(OH)D concentration was 8.48 nmol/L lower and odds ratio of vitamin D deficiency was 4.20 times (OR (95%CI): 4.20 (1.64, 10.77)) in children without 25(OH)D2 than those with 25(OH)D2 detected. Conclusions: Vitamin D deficiency was common among children aged 3–5 years in China. Vitamin D2 may play a role in preventing vitamin D deficiency in Chinese children aged 3–5 years.

## 1. Introduction

Vitamin D is a critical vitamin that regulates calcium, phosphorus [1] and bone metabolism [2]. Vitamin D deficiency early in life has been associated with decreased muscle strength [3], nutritional rickets, dental caries [4], sleep-disordered breathing [5], respiratory infections [6,7] and a more diverse lipid profile [8] in children. Higher serum 25(OH)D levels in youth have been associated with a reduced risk of developing type 2 diabetes mellitus [9], hypertension [10], cardiovascular disease [11] and cancer [12] later in adulthood.

Vitamin D insufficiency is common in China. The prevalence of vitamin D insufficiency (25(OH)D < 50 nmol/L) was reported to be 53.2% for Chinese children and adolescents aged 6–17 years in 2010–2012 [13]. Children aged 3–5 years are at the critical stage of growth. Currently, there is no nationwide prevalence on vitamin D deficiency for preschool children in China.

Vitamin D includes both vitamin D2 and vitamin D3. It is commonly considered that human vitamin D is mainly produced by skin through sunlight exposure, with a small amount coming through dietary intake, as it naturally is not present in many foods [14,15]. Sometimes vitamin D is even considered as a hormone rather than a nutrient. Vitamin D3 in the body comes from synthesis in the skin through sunlight exposure, nutrient supplementation and some animal source foods, such as fatty fish, fish liver oils and egg yolk [16]. Vitamin D2 in the body comes from nutrient supplements and dietary intake, mainly present in plants, plant products [17] and some meat of grazing animals [18]. Some research has found that vitamin D3 is more efficacious in raising total serum 25(OH)D concentration than vitamin D2 [19]. However, the evidence on how vitamins D2 contributes to vitamin D status is less clear. The aim of this study is to evaluate the vitamin D status of preschool children in China and to explore the role of vitamin D2 in vitamin D status.

## 2. Materials and Methods

### 2.1. Study Design

Data were collected from the Chinese National Nutrition and Health Surveillance in 2013 (CNNHS). The detailed methods of CNNHS 2013 were described previously [20,21]. Briefly, it was a cross-sectional survey with using a multi-stage stratified cluster sampling method. At the first stage, 2865 districts/counties from 30 provinces in China (Tibet autonomous region was not included) were classified into four categories, namely metropolises, medium and small cities, non-poor rural areas and poor rural areas. Then, 55 sites (12 metropolises, 15 medium and small cities, 18 non-poor rural areas, and 10 poor rural areas) were selected in the study based on proportion to population size. Then, 270 children aged 3–5 years were selected from each site, which was 90 in each age group (36–47 months, 48–59 months, and 60–71 months). A total of 14,850 children aged 3–5 years (36 to <72 months) were recruited, of whom at least 30 children at each site were sampled to assess their vitamin D status. The planned samples size for vitamin D was 1650. However, some parents refused to collect blood for children and some blood samples were not enough for vitamin D analysis.

### 2.2. Data Collection

The primary caregivers of children aged 3–5 years were interviewed using face-to-face interview by well-trained staff. A standardized questionnaire was used to collect the information of socioeconomic, family, lifestyle, health-related information, food frequency and supplement use information in the past week. A standard stadiometer and electronic weighting scale (Tc-200 K) were used to measure the height and weight respectively, without shoes, hat, heavy coat and untied hair.

### 2.3. Blood Sample and 25-Hydroxyvitamin D Measurement

Fasting venous blood (4 mL) was collected into vacutainer tubes (Becton Dickinson) containing potassium ethylene diamine tetraacetic acid (EDTA -K2), and then the samples were centrifuged at 1500× *g* for 15 min within 30–60 min. The serum used to test for vitamin D was aliquoted and stored in a brown centrifuge tube at −20~−80 °C in the local laboratory. All samples from the 55 study sites were transported to the National Institution for Nutrition and Health, China CDC by using cold chain. All the samples were preserved at −80 °C refrigerator until analysis. Serum 25(OH)D2 and 25(OH)D3 concentration were measured in qualified laboratory by liquid chromatography–mass spectrometry (LC-MS/MS, API 4000 Plus, AB SCIEX, USA). The LOD (limit of detection) of 25(OH)D2 and 25(OH)D3 were 2.45 nmol/L and 1.13 nmol/L, respectively. Both low and high concentration of quality control standards (RECIPE, Germany) of 25(OH)D2 and 25(OH)D3 were analyzed in every run. Inter-run coefficients of variations (CVs) for serum quality-control pools were 3.47% and 3.10% for low concentration and high concentration for 25(OH)D2 and 3.49% and 3.24% for low concentration and high concentration for 25(OH)D3, respectively.

### 2.4. Definitions of Study Variables

Total serum 25(OH)D concentration was the sum of serum 25(OH)D2 concentration and 25(OH)D3 concentration [22]. Total vitamin D concentration < 30 nmol/L is defined as vitamin D deficiency according to the Institution of Medicine (IOM) [23]. Based on the WHO growth standard in 2006 [24,25], wasting was defined as WHZ (weight-for-height Z-score) < −2, overweight was defined as 2 < WHZ ≤ 3, and obesity was defined as WHZ > 3, respectively. Urban areas included both metropolises and medium/small cities. Rural areas included both non-poor rural areas and poor rural areas. A family’s annual income per capita less than 15,000 CNY (Chinese Yuan) was defined as low income, 15,000–29,999 CNY as middle income, and more than 30,000 CNY as high income. Low latitude is defined as 20° N ≤ latitude < 30° N, middle latitude as 30° N ≤ latitude < 35° N, and high latitude as latitude ≥ 35° N [26,27]. The ethnicity was grouped according to their dressing habit. For example, the Muslin population have a covered clothing style, which may limit exposure of skin to natural light, whereas the Han population have an uncovered clothing style.

### 2.5. Statistical Analyses

Prevalence of vitamin D deficiency was presented as percentages (%(95% CI)) and concentration of 25(OH)D was described as median (P25,P75). Difference between 25(OH)D concentration was compared by Wilcoxon rank sum test or Kruskal–Wallis H rank sum test according to the classification of two or more groups. Chi-square test was used for categorical variables. Multiple linear regression was used to analyze the association between 25(OH)D concentration and potential related factors. Multiple logistic regression was performed to analyze associated risk factors for vitamin D deficiency. The variables included in the models were found to be significantly different in the concentration of vitamin D and vitamin D deficiency between different groups. The stepwise regression method was used for model selection (significance level for entry (SLE) = 0.20, significance level for stay (SLS) = 0.05). All statistical analyses were performed by SAS 9.4 software (SAS Institute Inc., Cary, NC, USA). The difference was considered as statistically significance with *p* < 0.05.

## 3. Results

### 3.1. Demographic Characteristics

In total, serum vitamin D was measured in 1481 children aged 3–5 years, 46 samples collected from April to September were excluded because the samples collected during these months were too scattered and the interaction between latitude and survey month could not be analyzed. Finally, 1435 children were included in the current study, 83.6% (1199/1435) of whom were Han ethnic and 2.2% (31/1435) of whom were Muslin ethnic. The percentage of subjects was similar across different latitude groups and almost all of field work was completed in fall/winter (from October to January) (Table 1).

### 3.2. Concentrations of Vitamin D and the Prevalence of Vitamin D Deficiency

Twenty-five hydroxy-vitamin D3 existed in all children’s blood sample, but 25(OH)D2 was only detected in 10.9% (156/1435) of the samples. The medians of serum 25(OH)D3 and total 25(OH)D for all children were 47.5 (37.5, 57.3) nmol/L and 49.3 (39.0, 58.5) nmol/L, respectively. The concentration of 25(OH)D2, 25(OH)D3 and total 25(OH)D were 10.1 (7.1, 16.0) nmol/L, 41.8 (38.3, 57.8) and 55.0(46.8, 65.8) nmol/L in the group with vitamin D2 detected, respectively. The prevalence of children suffered from vitamin D deficiency was 8.9%, which was 3.2% in the group with vitamin D2 detected and 9.5% in the group without vitamin D2 detected (Table 1).

### 3.3. Association of Vitamin D2 Detected and 25(OH)D Concentration

The associated factors of 25(OH)D concentration were shown in Table 2. Adjusted for age, sex, ethnic, residential area, outdoor time, combination of latitude and month studied, the expected 25(OH)D concentration was 8.48 nmol/L higher in the group with vitamin D2 detected than the group without vitamin D2 detected (*p* < 0.001).

### 3.4. Relationship between Vitamin D2 Detected and Vitamin D Deficiency

Influencing factors of vitamin D deficiency are shown in Table 3. The AUC (area under roc curve) of the multivariate logistic regression model was 0.81. Adjusted for age, sex, ethnic, residential area, combination of latitude and month studied, the children without 25(OH)D2 detected had a 4.20 (1.64, 10.77) times higher odds of vitamin D deficiency than those with 25(OH)D2 detected.

## 4. Discussion

The prevalence of vitamin D deficiency (25(OH)D < 30 nmol/L) was 8.9% overall and more than one-fifth of the children had vitamin D deficiency at latitude above 30° N in December. Children with detectable 25(OH)D2 had higher concentration of 25(OH)D and lower odds of vitamin D deficiency.

The prevalence of vitamin D deficiency among Chinese preschool children in this study was 8.9%, which was higher than 7.1% of children aged 1–5 years in NHANES 2011–2014 [28] and 7% of children aged 1–6 years old in Toronto (latitude 43.4° N) [29]. The prevalence of 25(OH)D < 50 nmol/L was 21.9% of children aged 2–5 years in a study from Hangzhou [30] and 15.1% of children aged 2–6 years in a study from Wenzhou [31], which are in southeastern China with low latitude and more seafood consumption. The prevalence of 25(OH)D < 50 nmol/L in Hangzhou of this study was significantly higher than that of children aged 2–5 years in winter and spring in the Hangzhou study (49% vs. <30%). The difference may be due to the fact that the subjects of the two studies were from different sources. The Hangzhou study was based on the children for health examination in Children’s Hospital Affiliated to Zhejiang University School of Medicine, while the present study was based on a population sample survey.

Vitamin D deficiency was more common at latitude above 30° N in November and December. However, vitamin D deficiency was not severe in October, even in the area of latitude above 35° N. At the areas of latitude below 30° N, vitamin D deficiency was not common from October to January. The prevalence of serum 25(OH)D < 25 nmol/L ranged from approximately <1–5% in the winter/lower latitude subpopulation (November–March, median latitude 32° N, range 25–41° N) and in summer higher latitude subpopulation (April–October, median latitude 39° N, range 25–47° N) of adolescents and adults from NHANES III [26]. It was consistent with other studies that strength of sunlight declines with increasing latitude and the sunshine time is longer in summer/fall than in winter/spring [32].

The prevalence of detectable 25(OH)D2 was 10.9% of children in the current study, which was similar to NHANES 2007–2010 (11% in children aged 1–5 years) [27]. In the National Adults Nutrition Survey (NANS) in Ireland with a low limit of quantification (1.43 nmol/L), 78.7% adults had serum 25(OH)D2. The median of 25(OH)D2 concentration (10.0 nmol/L) in our study was higher than the children age 1–5 years in NHANES (<5 nmol/L). The P90 value (6.36 nmol/L) and the P95 value (8.19 nmol/L) of 25(OH)D2 in NHANS were lower than the median of 10.0 nmol/L in the current study. Therefore, the rate of vitamin D2 detected was slightly lower due to the different LOD, but the concentration of vitamin D2 was higher in Chinese preschool children.

Adjusting for the effects of other possible factors, the group of 25(OH)D2 undetected was 4.20 times more likely to have vitamin D deficiency than the detected group. The median total 25(OH)D was significantly higher in the children with detectable vs. undetectable 25(OH)D2 (55.0 nmol/L vs. 48.3 nmol/L). These suggested that vitamin D2 played an important role in maintaining vitamin D status of Chinese children aged 3–5 years.

Unlike vitamin D3, vitamin D2 must be derived from dietary sources or nutrient supplements. It was generally considered that vitamin D2 was not commonly found in food. Due to the fact that vitamin D2 data are extremely sparce in food composition tables, it is difficult to calculate vitamin D2 intake and the contribution of vitamin D2 to total vitamin D. In the current study, the concentration of 25(OH)D2 was 10.1 (7.1, 16.0) nmol/L and total 25(OH)D was 55.0 (46.8, 65.8) nmol/L in the group with detected vitamin D2. In a study about osteoporotic fractures of men ≥65 years old, 25(OH)D2 (18.5 nmol/L) also played an important role to total 25(OH)D (64.5 nmol/L) in the group with detected 25(OH)D2 [33]. Vitamin D3 is more appropriate for supplementation or fortification in foods [34] because vitamin D2 might be less effective in raising levels of total 25(OH)D than vitamin D3 [35] and the poor stability of vitamin D2 is worrisome [36,37]. Many supplements are in the form of vitamin D3 and only high-dose, prescription vitamin D formulation is available for vitamin D2 in the United States [33]. In Europe, vitamin D2 is less frequently used for fortification and supplementation [38]. By searching for product information on the internet, the majority of vitamin D supplements on the China market are for vitamin D3. Thus, the serum vitamin D2 in the human body could mainly come from natural food.

Vitamin D2, which is converted by fungal sterol, ergosterol, mainly exists in plant foods. Simon et al. showed that mushrooms are excellent sources of vitamin D2 [39], and vitamin D2 baker’s yeast is produced by exposing baker’s yeast to ultraviolet light [40]. Mushrooms, fermented food and yeast are commonly consumed in China, which may be related to the high concentration of vitamin D2 in the current study. Interesting, unlike total vitamin D, the rate of vitamin D2 detected was higher in December than in October (not reported in the results), which needs to be further studied.

Our study has some limitations. The prevalence of vitamin D deficiency may be overestimated, as the survey was conducted from October to January, mainly in the winter. In addition, dietary vitamin D2 information was not available to assess its contribution; however, serum 25OHD2 might provide metabolized vitamin D2 contribution for Chinese children aged 3–5 years. Randomized control trials of dietary vitamin D2 intake study remain warranted to assess the effects.

## 5. Conclusions

Overall, vitamin D deficiency is relatively common for children aged 3–5 years in China, especially for those living above 30° N latitude in November and December, which could be a focus for vitamin D deficiency interventions. For a proportion of these children, dietary vitamin D2 might contribute to their vitamin D status and dietary vitamin D2 might be considered for improving vitamin D level of children in this age group in China. In the population with risk of vitamin D deficiency (e.g., high latitude, winter and/or indoor lifestyle), the magnitude of vitamin D2 intake contribution to overall vitamin D status warrants further studies by using longitudinal study designs.

## Figures and Tables

**Table 1:** Vitamin D status and prevalence of vitamin D deficiency of children by demographic characteristics in China.

	Characteristics (N)	Concentration Median (P25, P75) (nmol/L)				Vitamin D Deficiency *	
		# of 25(OH)D2 Detected	25(OH)D2	25(OH)D3	25(OH)D	# of Case	% (95% CI)
Total	1435	156	10.1 (7.1, 16.0)	47.5 (37.5, 57.3)	49.3 (39.0, 58.5)	127	8.9 (7.4, 10.3)
Vitamin D2 detected ^a, b^							
Yes	156	156	10.1 (7.1, 16.0)	41.8 (38.3, 57.8)	55.0 (46.8, 65.8)	5	3.2 (0.4, 6.0)
No	1279	0	0	48.3 (38.3, 57.8)	48.3 (38.3, 57.8)	122	9.5 (7.9, 11.2)
Age ^a^							
3 year	483	70	9.8 (7.5, 19.8)	51.0 (40.5, 59.8)	53.0 (43.3, 62.5)	35	7.3 (4.9, 9.6)
4 year	481	52	10.3 (6.9, 14.1)	46.3 (36.5, 55.3)	47.5 (37.5, 57.0)	48	10.0 (7.3, 12.7)
5 year	471	34	10.4 (7.3, 16.5)	45.5 (36.5, 56.0)	46.8 (37.5, 56.8)	44	9.3 (6.7, 12.0)
Sex ^a, b^							
Boy	720	82	9.8 (7.0, 16.3)	49.6 (38.8, 59.3)	51.3 (40.3, 60.8)	48	6.7 (4.8, 8.5)
Girl	715	74	10.8 (7.3, 15.8)	45.8 (36.5, 54.8)	47.3 (37.8, 55.5)	79	11.1 (8.8, 13.4)
Preterm or not							
No	1266	133	10.3 (7.0, 15.8)	47.5 (37.8, 57.0)	49.0 (39.3, 58.3)	111	8.8 (7.2, 10.3)
Yes	146	21	10.0 (7.3, 16.8)	47.0 (37.3, 58.3)	51.0 (39.3, 59.3)	15	10.3 (5.4, 15.2)
Ethnicity group ^a, b^							
Han	1199	132	10.4 (7.3, 16.4)	47.8 (38.0, 57.5)	49.5 (39.8, 58.5)	97	8.1 (6.6, 9.6)
Muslin	31	6	6.6 (6.3, 7.0)	36.0 (28.8, 45.3)	39.5 (28.8, 48.3)	9	29.0 (13.1, 45.0)
Others	204	18	10.3 (7.8, 17.0)	48.5 (35.6, 58.3)	49.1 (36.9, 59.3)	21	10.3 (6.1, 14.5)
Body weight status							
Wasting	18	3	8.0 (8.0, 11.3)	42.8 (34.3, 58.0)	42.9 (35.3, 58.3)	1	5.6 (0, 16.1)
Normal	1103	130	10.3 (7.3, 16.8)	47.5 (37.8, 57.5)	49.3 (39.5, 58.5)	96	8.7 (7.0–10.4)
Overweight	48	3	8.5 (5.8, 13.8)	43.9 (36.4, 54.1)	43.9 (36.4, 55.3)	8	16.7 (6.1–27.2)
Obesity	20	0	0	48.4 (40.1, 53.3)	48.4 (40.1, 53.3)	1	5.0 (0, 14.6)
Residential area ^a, b^							
Rural	717	67	10.5 (7.0, 15.8)	49.8 (40.3, 59.5)	50.8 (41.3, 61.0)	35	4.9 (3.3, 6.5)
Urban	718	89	9.8 (7.3, 16.8)	45.6 (35.3, 54.8)	47.3 (36.5, 56.3)	92	12.8 (10.4, 15.3)
Outdoor time (per day) ^a^							
≥2 h	1033	96	10.4 (7.0, 17.3)	48.8 (38.3, 58.3)	50.5 (40.0, 59.5)	86	8.3 (6.6, 10.0)
<2 h	395	60	9.9 (7.6, 13.8)	44.5 (35.0, 54.3)	47.0 (36.8, 55.8)	40	10.1 (7.2, 13.1)
Family income							
Low	746	75	10.8 (7.0, 18.3)	47.8 (38.3, 58.0)	49.0 (40.0, 59.0)	55	7.4 (5.5, 9.3)
Medium	292	30	8.5 (6.8, 12.8)	47.8 (37.8, 56.1)	49.3 (38.1, 58.3)	30	10.3 (6.8, 13.8)
High	255	29	9.5 (7.8, 15)	47.5 (37.0, 56.5)	49.8 (37.8, 58.0)	22	8.6 (5.2, 12.1)
Latitude and survey month ^a, b^							
High latitude, October	105	6	11.5 (7.5, 13.3)	50.0 (40.3, 57.8)	50.8 (42.3, 58.0)	5	4.8 (0.7, 8.8)
High latitude, November	174	17	8.0 (6.5, 11.0)	45.4 (37.0, 55.3)	47.6 (38.3, 56.0)	21	12.1 (7.2, 16.9)
High latitude, December	206	34	10.9 (8.0, 15.8)	36.3 (28.3, 47.3)	38.3 (30.0, 49.5)	51	24.8 (18.9, 30.7)
High latitude, January	17	0	0	56.5 (46.8, 62.8)	56.5 (46.8, 62.8)	0	0.0
Medium latitude, October	146	8	10.9 (7.5, 17.8)	50.3 (42.8, 58.3)	50.5 (42.8, 58.5)	3	2.1 (0, 4.4)
Medium latitude, November	198	26	8.0 (6.8, 12.8)	49.3 (38.8, 57.5)	51.0 (40.3, 59.5)	14	7.1 (3.5, 10.6)
Medium latitude, December	73	6	8.9 (7.3, 15.8)	41.0 (31.0, 50.8)	41.3 (32.0, 52.0)	16	21.9 (12.4, 31.4)
Medium latitude, January	31	3	8.0 (5.8, 8.3)	47.8 (40.3, 56.8)	48.8 (40.3, 56.8)	3	9.7 (0, 20.1)
Low latitude, October	38	3	9.3 (8.3, 32.5)	52.1 (42.3, 61.5)	55.6 (42.8, 64.8)	0	0.0
Low latitude, November	241	35	10.8 (7.0, 17.5)	51.3 (42.3, 60.5)	53.3 (43.5, 62.5)	7	2.9 (0.8, 5.0)
Low latitude, December	188	18	12.9 (8.0, 22.3)	50.3 (38.4, 61.9)	52.3 (41.4, 63.0)	7	3.7 (1.0, 6.4)
Low latitude, January	18	0	0	51.4 (41.0, 56.5)	51.4 (41.0, 56.5)	0	0.0

* Total vitamin D concentration < 30 nmol/L is defined as vitamin D deficiency. # number. Wilcoxon rank sum test or Kruskal–Wallis H rank sum test for comparing the difference of 25(OH)D concentration. Chi-square test for comparing the difference of 25(OH)D deficiency. ^a^ *p* < 0.05 (Wilcoxon rank sum test or Kruskal–Wallis H rank sum test). ^b^ *p* < 0.05 (chi-square test). CI, confidence interval.

**Table 2:** Adjusted association of vitamin D2 detected and 25(OH)D concentration of children aged 3–5 years in China.

Risk Factors	*n*	β (SE)	*p*
Vitamin D2 detected			
Yes	156	Ref.	-
No	1279	−8.48 (1.25)	<0.001
Age			
3 year	483	Ref.	-
4 year	481	−5.21 (0.94)	<0.001
5 year	471	−5.33 (0.96)	<0.001
Sex			
Boy	720	Ref.	-
Girl	715	−4.29 (0.77)	<0.001
Ethnicity group			
Han	1199	Ref.	-
Muslin	31	−7.01 (2.73)	0.010
Others	204	−2.81 (1.19)	0.019
Residential area			
Rural	717	Ref.	-
Urban	718	−3.17 (0.85)	<0.001
Outdoor time (per day)			
≥2 h	1033	Ref.	-
<2 h	395	−1.68 (0.90)	0.061
Latitude and survey month			
High latitude, October	105	8.62 (1.85)	<0.001
High latitude, November	174	8.03 (1.51)	<0.001
High latitude, December	206	Ref.	-
High latitude, January	17	11.41 (3.74)	0.002
Medium latitude, October	146	10.37 (1.67)	<0.001
Medium latitude, November	198	8.34 (1.51)	<0.001
Medium latitude, December	73	3.01 (2.02)	0.138
Medium latitude, January	31	14.69 (2.83)	<0.001
Low latitude, October	38	12.49 (2.63)	<0.001
Low latitude, November	241	12.97 (1.42)	<0.001
Low latitude, December	188	12.90 (1.53)	<0.001
Low latitude, January	18	10.63 (3.68)	0.004

Ref. = group of reference. Model: multiple linear regression model. Significant difference (*p* < 0.05).

**Table 3:** Relationship between vitamin D2 detected and vitamin D deficiency of children aged 3–5 years in China.

Risk Factors	Vitamin D Deficiency	OR (95% CI)	*p*
Vitamin D2 detected			
Yes	5/156	Ref.	-
No	122/1279	4.20 (1.64, 10.77)	0.003
Sex			
Boy	48/720	Ref.	-
Girl	79/715	2.17 (1.45, 3.26)	<0.001
Ethnicity group			
Han	97/1199	Ref.	-
Muslin	9/31	3.57 (1.48, 8.61)	0.005
Others	21/204	1.81 (1.01, 3.25)	0.048
Residential area			
Rural	35/717	Ref.	-
Urban	92/718	2.18 (1.38, 3.45)	<0.001
Latitude and survey month			
High latitude, October	5/105	0.23 (0.09, 0.64)	0.005
High latitude, November	21/174	0.39 (0.22, 0.69)	0.001
High latitude, December	51/206	Ref.	-
High latitude, January	0/17	-	-
Medium latitude, October	3/146	0.08 (0.02, 0.27)	<0.001
Medium latitude, November	14/198	0.29 (0.15, 0.56)	<0.001
Medium latitude, December	16/73	0.87 (0.44, 1.71)	0.678
Medium latitude, January	3/31	0.34 (0.10, 1.21)	0.095
Low latitude, October	0/38	-	-
Low latitude, November	7/241	0.09 (0.04, 0.20)	<0.001
Low latitude, December	7/188	0.12 (0.05, 0.29)	<0.001
Low latitude, January	0/18	-	-

Ref. = group of reference. Model: multiple linear regression model. Model’s AUC (area under roc curve) = 0.81. Significant difference (*p* < 0.05). CI, confidence interval.

## Data Availability

The datasets generated or analyzed during the current study are not publicly available due to the data management requirements of our institution, but are available from the corresponding author on reasonable request.

## Abbreviations

CNNHS	Chinese national nutrition and health surveillance
CNY	Chinese Yuan
CVs	coefficients of variations
IOM	institution of medicine
IQR	inter-quartile range
LC-MS	liquid chromatography- mass spectrometry
LOD	limit of detection
OR	Odd ratio
SLE	significance level for entry
SLS	significance level for stay
WHZ	weight-for-height Z-score
95% CI	95% confidence interval
